# Draft Genome Sequences of Three Hypervirulent Carbapenem-Resistant *Klebsiella pneumoniae* Isolates from Bacteremia

**DOI:** 10.1128/genomeA.01081-16

**Published:** 2016-12-08

**Authors:** Chaitra Shankar, Laura E. B. Nabarro, Naveen Kumar Devanga Ragupathi, Dhiviya Prabaa Muthuirulandi Sethuvel, Jones Lionel Kumar Daniel, George Priya Doss C, Balaji Veeraraghavan

**Affiliations:** a Department of Clinical Microbiology, Christian Medical College, Vellore, Tamil Nadu, India; b School of Bio Sciences and Technology, VIT University, Vellore, Tamil Nadu, India

## Abstract

Hypervirulent *Klebsiella pneumoniae* strains have been increasingly reported worldwide, and there is emergence of carbapenem resistance among them. Here, we report the genome sequences of three carbapenem-resistant hypervirulent *K. pneumoniae* isolates isolated from bacteremic patients at a tertiary-care center in South India.

## GENOME ANNOUNCEMENT

*Klebsiella pneumoniae* is a common pathogen causing both community-acquired and hospital-acquired infections (1). Carbapenems are the drug of choice for treating extended-spectrum β-lactamase (ESBL)-producing *K. pneumoniae* infections. However, carbapenem resistance is now commonly seen due to increased usage and leads to a high mortality rate of 30% to 44% (2–4). Hence, tigecycline and colistin are current therapeutic choices.

Hypervirulent *K. pneumoniae*, first identified from cases of liver abscess, has been increasingly reported worldwide. *K. pneumoniae* strains have characteristic hypermucoviscous colonies on culture medium, identified phenotypically by a positive string test. They are associated with several virulence factors compared to the classical strains, which include *rmpA, rmpA2, magA*, siderophores, such as aerobactin, enterobactin, and yersiniabactin, and genes coding for allantoin metabolism.

Three string test-positive carbapenem-resistant *K. pneumoniae* isolates (B1647, B20038, and B20143) from blood culture were identified. All three isolates were from health care-acquired infections, and two of the three patients died. The meropenem MICs for the isolates were >16 µg/ml, as determined by Etest (bioMérieux). DNA for the isolates were extracted using QIAsymphony, as per the manufacturer’s instructions. Whole-genome sequencing was performed by next-generation sequencing using IonTorrent and assembled using SPAdes version 5.0. The contigs were annotated using RAST (http://rast.nmpdr.org/) and Patric (http://patricbrc.org/). The MLST, ResFinder, and PlasmidFinder (http://www.genomicepidemiology.org/) databases were used to find the sequence type, antibiotic resistance genes, and the plasmid types present in the isolates. Virulence genes were defined with the help of database available at http://bigsdb.pasteur.fr/perl/bigsdb/bigsdb.pl?db=pubmlst_klebsiella_seqdef_public&page=downloadAlleles.

The isolates were found to harbor the *rmpA2* gene, which is characteristically associated with hypervirulent strains. The isolates belonged to three different sequence types (STs), namely, ST11, ST43, and ST231, indicating that they are from different lineages. The isolates do not belong to the K1 serotype, as they lacked the *magA* gene. The isolates harbored IncFIA, IncFIB, IncFII, IncHI1B, and Col types of plasmids. Since the isolates were multidrug resistant, they were found to harbor several genes coding for antimicrobial resistance against aminoglycosides, quinolones, beta-lactams, fosfomycin, and rifampin. *aac(6′)lb-cr*, coding for both fluoroquinolones and aminoglycosides, was also found. B1647 and B20143 showed the presence of genes coding for phenicol and sulfonamide resistance. All three isolates harbored *bla*_OXA_ genes (*bla*_OXA-232_, *bla*_OXA-181_, and *bla*_OXA-1_), while the third isolate, B20143, had both the *bla*_NDM-1_ and *bla*_OXA-1_ genes. Among the genes encoding carbapenem resistance, *bla*_NDM_ and *bla*_OXA_ genes are commonly seen in India (5, 6). The isolates encoded genes for siderophores, such as *iutA, iucA, iucB,* and *iucD* for aerobactin; *entA, entD, entE,* and *entF* for enterobactin, and *ybtA, ybtU, ybtT, irp1, irp2,* and *fyuA* for yersiniabactin. Siderophores, one of the important virulence factors, have varied affinities for iron, with aerobactin having the lowest affinity and enterobactin having the highest (7, 8). B1647 and B20143 isolates carried the *mrkD* gene encoding type 3 fimbriae, which aids biofilm formation (9). All carried the virulence factor MviM and genes coding for iron acquisition, such as *kfuA, kfuB,* and *kfuC*.

### Accession number(s).

The whole-genome sequences of the three carbapenem-resistant hypervirulent *K. pneumoniae* (CR-hvKp) isolates have been deposited at GenBank under the accession numbers MCFO00000000 (B1647), MCFP00000000 (B20038), and MCFQ00000000 (B20143).
